# A survey of elastase-producing bacteria and characteristics of the most potent producer, *Priestia megaterium* gasm32

**DOI:** 10.1371/journal.pone.0282963

**Published:** 2023-03-13

**Authors:** Ghadah A. AlShaikh-Mubarak, Essam Kotb, Amira H. Alabdalall, Munirah F. Aldayel

**Affiliations:** 1 Basic and Applied Scientific Research Center (BASRC), Imam Abdulrahman Bin Faisal University (IAU), Dammam, Saudi Arabia; 2 Department of Biology, College of Science, Imam Abdulrahman Bin Faisal University (IAU), Dammam, Saudi Arabia; 3 Department of Biological Sciences, College of Science, King Faisal University, Al Ahsa, Saudi Arabia; King Saud University, SAUDI ARABIA

## Abstract

Ninety-one elastase-producing bacterial isolates were recovered from different localities of the Eastern Province of Saudi Arabia. Elastase from the best isolate *Priestia megaterium* gasm32, from luncheon samples was purified to electrophoretic homogeneity using DEAE-Sepharose CL-6B and Sephadex G-100 chromatographic techniques. The recovery was 17.7%, the purification fold was 11.7*x*, and the molecular mass was 30 kDa. Enzymatic activity was highly repressed by Ba^2+^ and almost completely lost by EDTA, but it was greatly stimulated by Cu^2+^ ions, suggesting a metalloprotease type. The enzyme was stable at 45°C and pH 6.0–10.0 for 2 hours. Ca^2+^ ions considerably enhanced the stability of the heat-treated enzyme. The *V*_max_ and *K*_m_ against the synthetic substrate elastin–Congo red were 6.03 mg/mL, and 8.82 U/mg, respectively. Interestingly, the enzyme showed potent antibacterial activity against many bacterial pathogens. Under SEM, most bacterial cells showed loss of integrity, damage, and perforation. SEM micrographs also showed a time-dependent gradual breakdown of elastin fibers exposed to elastase. After 3 hours, intact elastin fibers disappeared, leaving irregular pieces. Given these good features, this elastase may be a promising candidate for treating damaged skin fibers with the inhibition of contaminating bacteria.

## Introduction

Elastin is a basic matrix component responsible for tissue flexibility. It is found in the skin, arteries, lungs, and other tissues. Depending on the tissue type, elastin is present in variable amounts, forming fibers of extensively cross-linked protein. It is insoluble and has a high degree of hydrophobicity. These characteristics are relatively stable and durable in tissues unless one or more proteinases degrades the elastin fibers [1].

Elastases are special types of proteolytic enzymes that can degrade elastin as a specific substrate. They are subdivided into different groups of proteases, particularly serine proteases, aspartic proteases, thiol proteases, and metalloenzymes [2]. They are different in their catalysis and substrate specificity [1]. They were initially discovered in the viscera of animals. Nonetheless, this source is insufficient to meet the needs of industry and daily use. Microorganisms can produce high levels of elastases [3].

For several years, bacterial elastases were classified as metalloproteinases, which require Zn^2+^ ions as cofactors for their activity. *Streptomyces fradiae* and *Bacillus thermoproteolyticus* elastases are among the most potent elastolytic proteinases discovered to date because they are 4-8-fold more effective than pancreatic elastases [4].

The requirement for unique eco-friendly bacterial-derived elastases that can be used in various industrial and pharmaceutical applications has increased. They have widespread usage in food processing, medical treatment, and the chemical industry [5]. Additionally, elastases immobilized on a bandage are used to treat various ailments, including carbuncles, damaged skin, furuncles, and burns [6].

The majority of microbial elastases are produced by *Pseudomonas aeruginosa* [7], *Staphylococcus epidermidis* [8], *Bacillus licheniformis* [5], *Bacillus subtilis* [9], and *Chryseobacterium indologenes* [3]. Unfortunately, most of them have low stability under environmental conditions.

Antimicrobial resistance is wreaking havoc on people’s health. As a result, ten million people have died worldwide [10]. In this research, we aimed to produce a highly stable elastase that can hydrolyze elastin fibers efficiently while also having antibacterial activity. Therefore, it can be applied in the treatment of damaged skin and the targeting of contaminating bacteria.

## Materials and methods

### Materials

Elastin–Congo red, azocasein, and EDTA were purchased from Sigma‒Aldrich (USA). Sephadex G-100 FF, DEAE-Sepharose CL-6B, and SDS‒-PAGE reagents were procured from Pharmacia Biotech (Sweden). The other materials were sourced from regional providers.

### Strain isolation

Throughout this inspection, twenty samples of seawater and soil were collected from Tarout Island (26° 33’ 59.99" N, 50° 03’ 60.00" E), Al Khobar city (26° 13’ 1.8876’’ N, 50° 11’ 49.6968’’ E), and Al Ahsa city (25° 25’ 27.59" N, 49° 37’ 11.39" E) in the Eastern Province of Saudi Arabia from August to November 2019. Additionally, we collected five samples of crude camel milk, five shrimp shell waste samples, five crude cheese samples, and five processed meat samples from the Al Ahsa public market. Protease production was detected by growing isolates on 25% (v/v) skimmed-milk agar containing (gr/L) agar (15), NaCl (5), beef extract (1) peptone (5), and yeast extract (2). The medium pH was adjusted to 7.0, and incubation was performed at 37°C for 24 hours to visualize the positive hydrolytic colonies. For further experiments, pure cultures were stored at -80°C in 20% glycerol.

### Characterization of the most potent isolates at the species level

Extraction of DNA from the bacterial cells was performed according to Dellaporta et al. [11] as follows: 20 mg of freshly harvested cells was broken up with 0.5 mL of Dellaporta buffer (100 mM Tris-HCl pH 8, 500 mM NaCl, 50 mM ethylenediaminetetraacetate (EDTA), 10 mM β-mercaptoethanol). Specifically, 33 μL of 20% sodium dodecyl sulfate (SDS, w/v) was added to the cells and they were vortexed and incubated at 65°C for 10 min. Then, 160 μL of 5 M potassium acetate was added, and the mixture was vortexed, followed by centrifugation for 10 min at 10,000 rpm. The supernatant was collected, and 450 μL of it was combined with the same volume of phenol, chloroform, and isoamyl-alcohol mixture at a ratio of 25:24:1. The mixture was vortexed for 5 min prior to centrifugation at 10,000 rpm for 5 min. Then, 400 μL of the upper phase was removed, mixed with a half volume of isopropanol, vortexed, and centrifuged at 14,000 rpm for 10 min. The nucleic acid precipitate was cleaned with 70% ethanol and centrifuged at 10,000 rpm for 5 min. The precipitate was finally resuspended in 100 μL of distilled water.

Amplification of the 16S rRNA gene was performed using the universal primers 1492R 5′-TACGGYTACCTTGTTACGACTT and 27F 5′-AGAGTT TGATCMTGGCTCAG-3′ [12]. The main steps of polymerase chain reaction (PCR) were as follows: 94°C for 5 min, denaturation at 94°C for 45 sec, annealing at 55°C for 60 sec, and extension at 72°C for 60 sec. After thirty cycles, the last extension was performed at 72°C for 10 min (Applied Biosystems™ Veriti™ Thermal Cycler, 96-Well, USA). The QIAquick^®^ PCR Purification Kit was employed to purify the PCR products according to the manufacturer’s guidelines. The DNA sequences in both directions were then determined at Macrogen Inc. (Korea) and uploaded to Blastn of GenBank and a similarity check was run. For phylogenetic inference analysis, the closely related sequences were used alongside the sequences retrieved for all bacteria to establish phylogenetic trees using MEGA11 software. Information on the phylogenetic tree is available in S3 Fig.

### Enzyme production and protein measurement

The crude enzyme was produced in a minimal medium composed of (%, w/v) peptone (0.5), fructose (0.5), KH_2_PO_4_ (0.08), NaCl (0.3), yeast extract (0.04), MgSO_4_.7H_2_O (0.05), K_2_HPO_4_ (0.02), CaCl_2_ (0.01), MnSO_4_ (0.01), FeSO_4_ (0.002) and ZnSO_4_ (0.002). The medium pH was adjusted to 7.0 and then dispensed at 20% (v/v) in 250 mL Erlenmeyer flasks. A2% (v/v) inoculum of 12-hour-old cultures (1.0×10^8^ cfu/mL) in LB broth was used. LB consisted of (%, w/v) tryptone (1.0), NaCl (0.5), and yeast extract (0.5) at pH 7.0. Fermentation was allowed for 48 hours at 37°C with shaking at 200 rpm. Centrifugation was performed at 5,000 rpm for 20 min using a refrigerated centrifuge (Hermle Labortechnik GmbH, Z 326 K, Germany) at 4°C to separate the bacterial cells. Supernatants were used for the determination of elastase productivity and protein concentration as described by Kotb et al. [13] with slight modifications.

The amount of protein was quantified by measuring the absorbance of the culture filtrate at 280 nm. The optical densities were converted to weights from the standard curve of varying concentrations of bovine serum albumin (BSA).

### Enzyme assays

Dual plate assays: A preliminary protease assay was performed on Petri dishes containing 2% (w/v) skimmed milk and 1.5% (w/v) agar [14]. In parallel, an elastase assay was performed in nutrient agar plates supplemented with elastin–Congo red as a substrate [15]. Isolates were checked for enzyme productivity using spotting and well plate techniques. After creating wells with a diameter of 7 mm on Petri dishes, 0.1 mL of crude enzymes was introduced to every well. Petri dishes were then incubated at 37°C for 24 hours. Zones of substrate hydrolysis were used as a measure of enzyme productivity.

Protease assay: Cultures of the ninety-one bacterial isolates were shake-incubated at 37°C for 48 hours in minimal medium to allow enzyme productivity. The cell-free supernatants (CFS) were obtained after centrifugation at 5,000 rpm for 20 min using a cooling centrifuge at 4°C. The reactants were 0.5 mL of 0.08% (w/v) azocasein solution as a substrate and 0.5 mL of CFS. The azocasein was dissolved in 0.2 M potassium phosphate buffer at pH 7.0. Then, the mixture was incubated at 37°C for 30 min, and 1 mL of 10% (w/v) trichloroacetic acid (TCA) was added. After incubation in a crushed ice bath for 1 hour, the unreacted azocasein was precipitated by centrifugation. An equal volume of supernatant and NaOH (1 N) were mixed, and the absorbances were measured at 440 nm [16, 17]. A blank was prepared by the same procedure, replacing the enzyme with distilled water. Under usual test conditions, one unit (**U**) of enzyme activity was defined as the quantity of enzyme that produced an increase in 0.01 absorbance at 440 nm per minute. The tests were performed in triplicate, and the mean values were recorded as units of protease activity.

Elastase assay: Every 1 mL of elastase was mixed with 20 mg of the substrate Congo red elastin suspended in 2 mL of 0.2 mol/l acid buffer (pH 7.4) with shake incubation at 37°C for 20 min. The reaction was ended by adding 100 μL of 0.12 M EDTA (pH 8.0). Following centrifugation, the absorbance at 495 nm was calculated with a spectrophotometer calibrated to a control elastin–Congo red sample incubated without the enzyme [18]. One unit (**U**) of elastase was equivalent to the quantity of enzyme causing a rise in *A*_495_ by 0.01 after 1 hour of incubation under the standard assay conditions.

### Enzyme purification

The elastolytic enzyme from the most potent isolate was purified through 3 main stages: fractional precipitation with (NH_4_)_2_SO_4_, DEAE-Sepharose CL-6B anion-exchange, and Sephadex G100 FF gel permeation chromatography. Bacteria separated from the fermentation medium by centrifugation at 5000 rpm for 15 min. (NH_4_)_2_SO_4_ crystals were added slowly to the crude enzyme preparation until 70% saturation was reached. Agglomerated enzyme molecules were collected by centrifugation (7,*000 × g* for 10 min, 4°C) after overnight incubation at 4°C. The pellet was mixed with the least volume of 100 mM phosphate buffer (pH 7.4, buffer A) necessary for resuspension. Dialysis was then performed against buffer A to remove ammonium sulfate and other undesirable salts. The concentrated dialysate was then applied onto a DEAE-Sepharose CL-6B column (0.5×5 cm^2^) equilibrated with buffer A. A linear rise of 0–1 M NaCl in 0.1 M borate buffer (pH 9.4, buffer B) was used as an eluent. Fractions showing elastase activity were collected and concentrated for application through a second Sephadex G100 FF column (2.0 × 45 cm^2^). The elution rate was adjusted to 1 mL/3 min. The elastase active fractions were merged and lyophilized for characterization studies. To examine the homogeneity and molecular mass of the target elastase, SDS‒PAGE analysis was performed with a 5% (w/v) stacking gel and a 15% (w/v) separating gel [19].

### Effect of pH on enzymatic activity and stability

Enzyme activity was tested at pH values of 4 to 12 in a suitable buffer by incubating crude enzyme substrate under standard assay conditions. Experiments were carried out utilizing the typical enzyme assay with the substrate azocasein (0.8%, w/v) with incubation at 45°C for 30 min based on the result of the optimal temperature for maximal enzyme activity, which was 45°C. As previously stated, relative activity was determined. For the pH stability test, buffered enzyme fractions were preincubated at pH 4–12 for 2 hours at 45°C. The residual activity against the substrate was then assayed at pH 8.0 and 45°C.

### Effect of temperature on the enzymatic activity and stability

The optimal temperature for elastase activity was determined by mixing elastase with its substrate at a ratio of 1:1 and incubating at 20–50°C for 30 min. The relative elastase activities were determined as the percentage of the maximum activity (100%) under the standard assay conditions.

### *Relative activity = [Total activity (U/mL) × 100] ÷ Maximum activity (U/mL)* [20]

A thermal stability assay was designed to determine the temperature range within which the tested elastase maintained its activity. The enzyme preparation was incubated alone in a water bath for 15, 30, 45, and 60 min at 50, 60, 70, and 80°C. The residual elastase activity was assessed against the substrate at 45°C for 30 min as previously mentioned.

The impact of calcium ions on thermostability was determined by preincubating the elastase preparation at 60°C in the presence of CaCl_2_. Calcium chloride was tested at concentrations of 2, 5, 10, and 15 mM. The remaining activity was then determined after 60 min of heat exposure. The nonheated enzyme was considered a control and expressed as 100% activity.

### Effect of metallic ions and elastase inhibitors

The effect of various cations, such as Co^2+^, Ba^2+^, Mg^2+^, Ca^2+^, Hg^2+^, Fe^3+^, Na^+^, Mn^2+^, Fe^2+^, Mg^2+^, Cu^2+^, and Zn^2+^on elastase activity was examined. The enzyme was incubated at 35°C for 2 hours with metallic ions at a concentration of 5 mM.

The mixture was then incubated with 5.0 mL of 0.08% (w/v) azocasein as a substrate in 200 mM potassium phosphate buffer (pH 7.0) at 45°C for another 2 hours.

For the inhibition studies, the enzyme was preincubated for 2 hours with two concentrations of EDTA (5 and 10 mM) at room temperature, and then the residual activity was measured under the optimum assay conditions. The activity of the enzyme in the absence of metal ions and inhibitors defined as 100% activity.

### Kinetic parameters

The dynamics of elastase were tested against the substrates azocasein and elastin–Congo red. The reaction rate was assessed at 45°C and pH 8.0 thereafter, and the enzymatic activity was determined as described previously. The Michaelis–Menten constant (*K*_m_) and the maximum reaction velocity (*V*_max_) were then calculated from the linear regression equation created from the Lineweaver–Burk plot (LB plot). The turnover number (*K*_cat_) was determined by dividing the value of *V*_max_ by the adopted elastase concentration.

### Antibacterial activity of elastase

The antibacterial potential of the tested elastase was evaluated against several strains of bacterial pathogens, including *Escherichia coli*, *Salmonella* spp., *S*. *aureus*, *Shigella boydii* ATCC 9207, and *P*. *aeruginosa* ATTC 27853. The agar well diffusion method was adopted as illustrated by Khan et al. [21]. The agar layers were swabbed with bacteria at 1.0×10^8^ colony forming units/mL of physiological saline. Then, 6-mm diameter holes were pierced off aseptically with a sterilized cork borer. Each well received a certain concentration of the purified enzyme (100 U, 50 U, and 25 U) in a total volume of 100 μl, and then incubation was performed at 37°C for 24 hours. The inhibition areas around the wells were taken as direct measurements of the antibacterial activity.

The exact MIC values against the most affected bacteria *S*. *boydii* ATCC 9207 and *S*. *aureus* subsp. aureus Rosenbach ATCC 25923 were measured by the broth dilution method with few modifications [22]. An initial stock preparation of 4 mL for the tested elastase was adjusted at 100 U/mL in Muller Hinton Broth (MHB). Then, two-fold dilutions were made in 2 mL MHB tubes till reaching 6.25 U/mL concentration of the enzyme. Exactly, 200 μL from a bacterial culture suspension prepared in physiological saline at 1.0×10^8^ colony forming units/mL was inoculated into each broth culture. A broth culture with no bacteria served as the negative control (0% growth) while, a broth tube not involving the tested hydrolytic enzyme served as the positive control (100% growth). Incubation was done for 24 hours at 37°C. MIC was defined as the mean of the lowest concentration inhibiting bacteria and the highest concentration allowing the growth of bacteria.

### Scanning electron microscopy (SEM) examination

TESCAN VEGA3 SEM was used to observe the staphylolytic activity and shigellalytic activity of the target elastase on the exterior features of cells. *S*. *aureus* subsp. *aureus* Rosenbach ATCC 25923 and *S*. *boydii* ATCC 9207 were cultivated in LB broth for 24 hours at 37°C. Bacterial cells were harvested, and a fraction was suspended in an enzyme preparation for 20 hours at 37°C. A second fraction served as a blank control. Cells were collected at 5000 rpm for 20 min, rinsed two times in 0.2 M sodium phosphate buffer, pH 7.5, and loaded on adhesive SEM stubs for sample processing. Dehydration was performed with 30%-95% ethanol, and then the cells were incubated at 35°C for 2 hours to allow them to dry completely. Sputter coating with chromium for 15 min was performed to enhance the electron conductivity on the sample surfaces. Finally, TESCAN VEGA3 SEM was used to inspect the exterior shape of both sets at a low voltage of 20 keV.

*In vitro* application of elastase in the degradation of elastin fibers

Exactly, 0.5 mg of elastin fibers were mixed with one mL of elastase preparation in 50 mM Tris-HCl (pH 8.0) buffer and incubated for 3 hours at 37°C. The elastin fibers were separated by natural settling in an ice bath for 1 hour. This procedure was performed twice in physiological saline to clean the elastin fibers thoroughly. Elastin fibers in 50 mM Tris-HCl (pH 8.0) without elastase were utilized as a blank control. The fiber status was observed each hour [23] under LM and SEM. For SEM analysis, the fiber fractions were lyophilized before being placed on the SEM metal stub, coated with a 5 nm thick chromium layer and investigated with a Tescan VEGA3 SEM at 5.0 kV [24].

### Statistical analysis

SPSS Statistics Ver. 20.1 (IBM^®^ SPSS^®^ Statistics, Armonk, NY, USA) was employed to analyze the raw results. *P* values of less than 0.05 were judged statistically significant. Unless otherwise indicated, all data are expressed as the mean ± standard deviation (SD) of three repeats.

## Results

### Strain isolation

In the present study, ninety-one proteolytic isolates were recovered from different localities of the Eastern Province of Saudi Arabia. Clear zones around growing colonies were considered a positive indication of enzyme productivity. Isolates 25, 27, 28, 32, 34, 37, 43, 71, 82, and 91 were the highest producers of proteases and elastases on skimmed milk agar and elastin–Congo red agar. Isolate gasm32 displayed the highest proteolytic and elastolytic activity compared to the others (S1 Fig). Therefore, it was chosen for further research.

### Molecular characterization and phylogenetic inference of the most potent isolates at the species level

All DNA samples extracted with the QIAGEN Chromosomal kit were subjected to PCR (S2 Fig). The amplified PCR products were electrophoresed on a 1% (w/v) agarose gel. The extracted DNA appeared as single bands on the agarose gel after electrophoresis, confirming its purity. The partial *16S rRNA* sequences of all elastase-producing bacteria were compared with those of other bacterial strains deposited in GenBank using the similarity matrix, which was determined by the number of base differences (Table 1). The neighbor-joining phylogenetic tree constructed with MEGA11 software revealed that the gasm32 isolate recovered from the luncheon sample was a strain of *Priestia megaterium*, as it was clustered with *P*. *megaterium* strains (S3 Fig).

**Table 1:** The top ten elastase-positive bacterial isolates identified by 16S rRNA analysis.

Isolate	Source	Accession No.	Closest relative	Elastase productivity (U/mL)
*Priestia megaterium* gasm32	Luncheon	MW940856	*Priestia megaterium* strain 273	771.3 ± 2.0
*Bacillus aryabhattai* gasm34	Sausage	MW940858	*Bacillus aryabhattai* strain BCN4-1	723.1 ± 1.4
*Klebsiella pneumoniae* gasm37	Sausage	MW940939	*Klebsiella pneumoniae* strain AR_0158	721.9 ± 1.3
*Serratia marcescens* gasm82	Beach sand	MW940940	*Serratia marcescens* strain vaj5	453.2 ± 1.9
*Serratia marcescens* gasm91	Arabian Gulf water	MW940941	*Serratia marcescens* strain kal8	405.5 ± 2.1
*Macrococcus caseolyticus* gasm25	Cheese	MW940943	*Macrococcus caseolyticus* strain 190311L253	391.6 ± 1.4
*Proteus mirabilis* gasm43	Camel milk	MW940942	*Proteus mirabilis* strain abd2	724.3 ± 1.6
*Proteus* sp. gasm71	*Jasminum sambac* tree soil	MW940944	*Proteus* sp. strain KR 249	723.1 ± 1.2
*Enterobacter cloacae* gasm27	Luncheon	MW940945	*Enterobacter cloacae* strain Remi_3	385.1 ± 0.8
*Lactococcus lactis* gasm28	Luncheon	MW940946	*Lactococcus lactis* strain 4355	391.8 ± 0.5

### Enzyme purification

**Fig 1:**
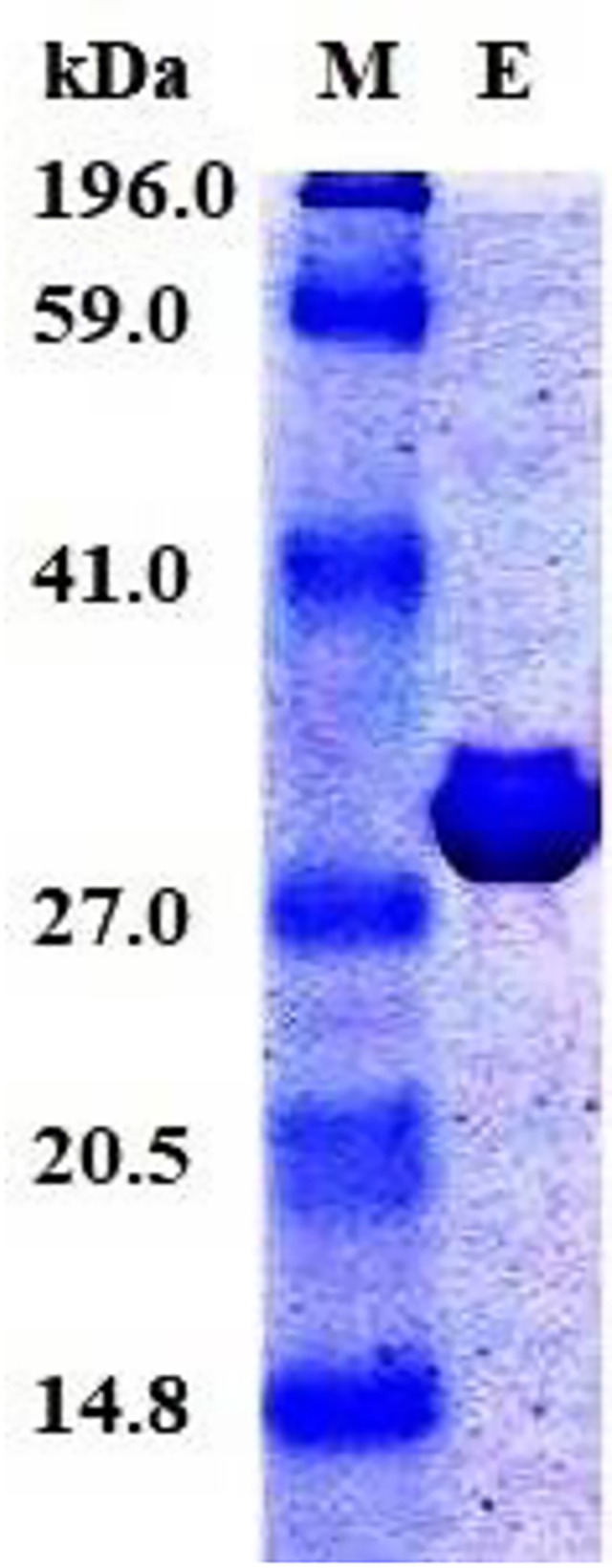
SDS‒PAGE of the purified elastase using a 5% stacking gel and a 15% separating gel.

Gasm32 elastase was separated from the broth of *Priestia megaterium* gasm32 using several procedures described in Table 2. The final specific activity of elastase reached 11.7-fold with a recovery of 17.7% compared with the initial concentration of elastase in the CFS of *P*. *megaterium* gasm32. SDS‒PAGE of the final elastase-active fractions revealed one band at 30 kDa (Fig 1). The previous version of the SDS‒PAGE gel is presented in S4 Fig.

**Table 2:** Summary of elastase purification steps.

Purification step	Total activity (U)	Specific activity (U/mg)	Recovery (%)	Purification (fold)
Cell free supernatant	5215.2	16.0	100.00	1.00
Ammonium sulfate precipitation	3548.4	37.4	68.04	2.34
Anion exchanger (DEAE-Sepharose CL-6B)	1725.7	91.7	33.09	5.73
Gel permeation (Sephadex G-100 FF)	924.8	186.5	17.73	11.66

### Effect of pH value on enzymatic reactivity and stability

**Fig 2:**
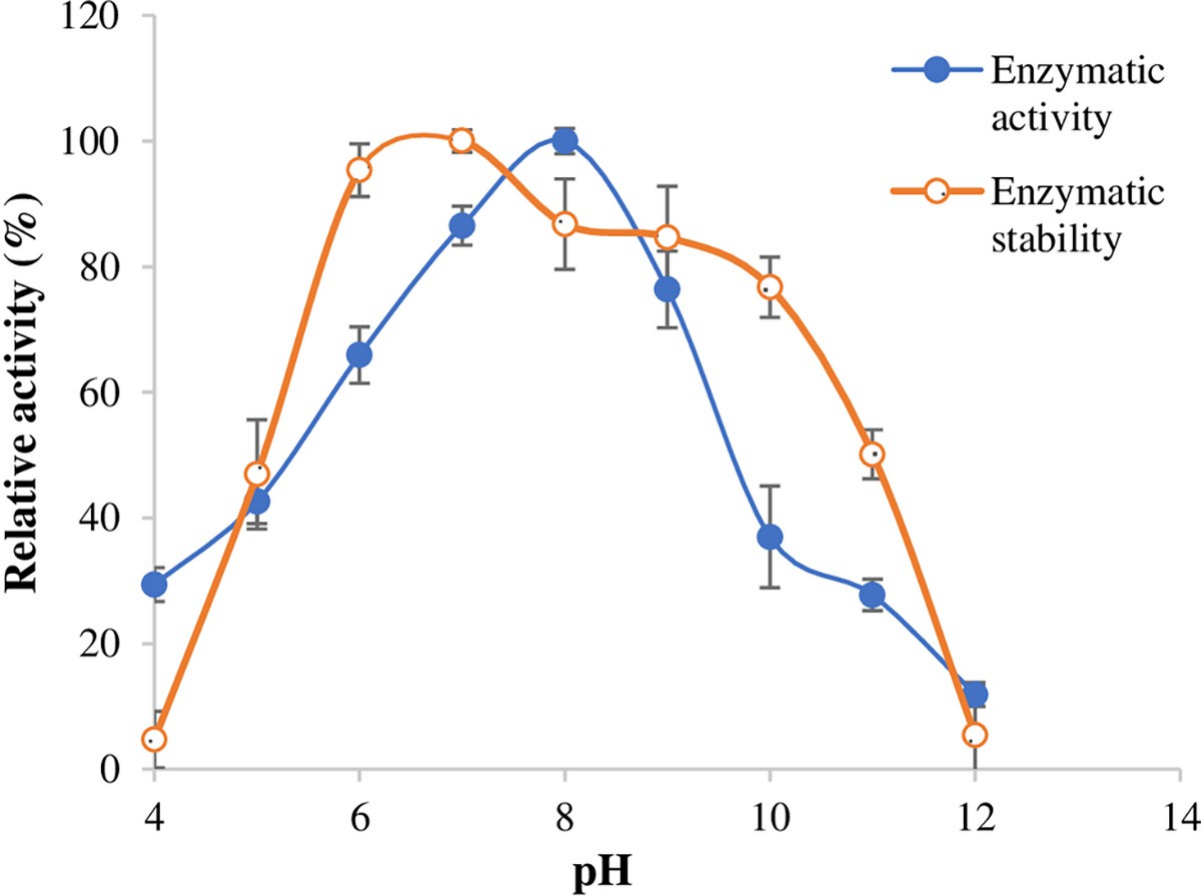
Relation of pH values to elastase activity and stability.

The results in Fig 2 indicated that the maximum reaction activity for elastase was at pH 8.0. In addition, the enzyme was stable at pH 6–10 for 2 hours. Above or below this particular pH range, the elastase stability drastically decreased.

### Effect of temperature on elastase activity and stability

**Fig 3:**
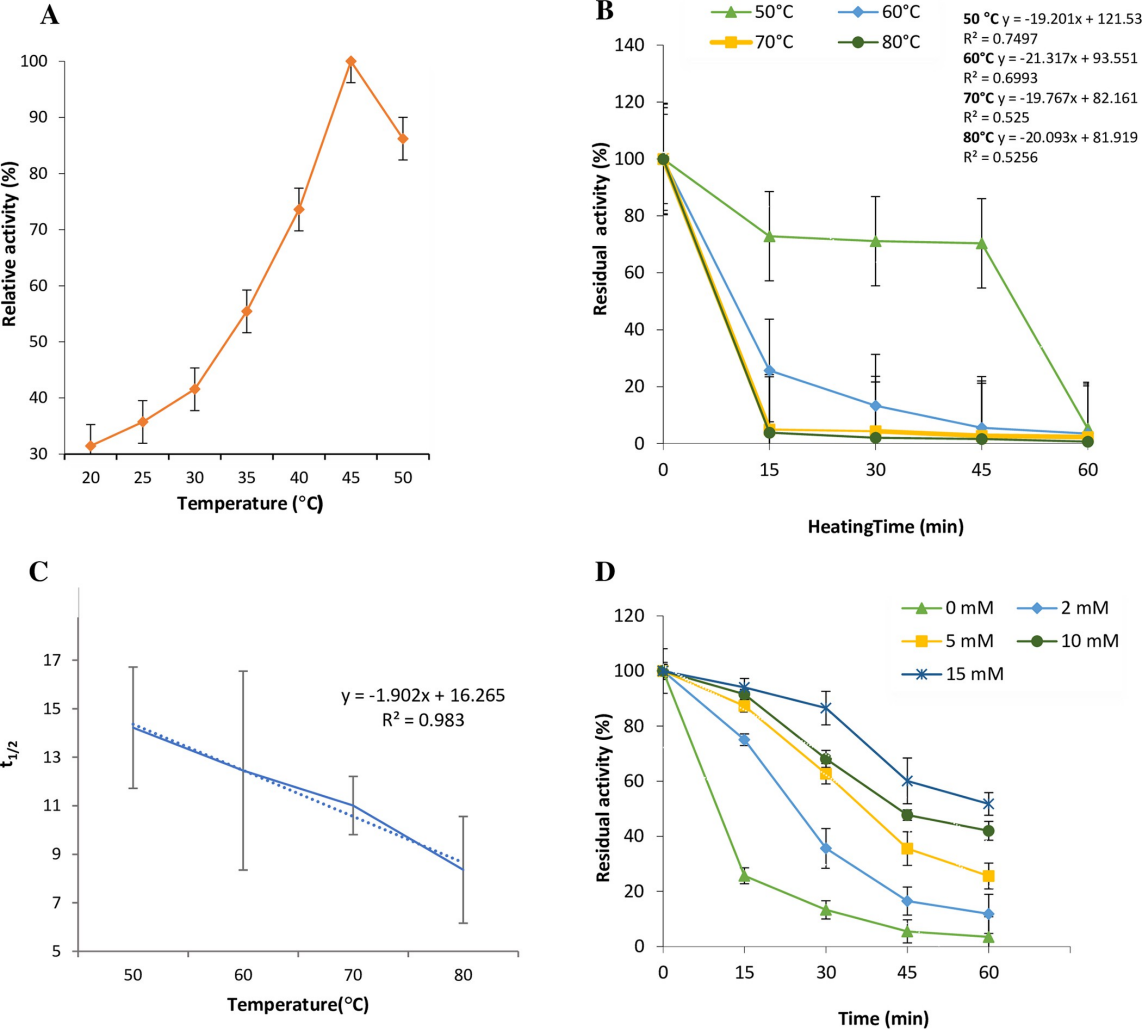
Relation of reaction temperatures to elastase activity (A) and stability (B). The calculated half-lives (t_1/2_) are presented in Panel C and the effect of calcium ions on elastase thermostability is shown in Panel D.

The optimal temperature for maximal elastase activity was observed at 45°C (Fig 3A). The results in Fig 3B revealed that the enzyme was heat-stable up to 60°C for 15 min. The half- lives (t_1/2_) were 14.22 min, 12.45 min, 11.01 min, and 8.36 min at 50°C, 60°C, 70°C, and 80°C, respectively (Fig 3C). As shown in Fig 3D, the enzyme stability was substantially improved by addition of Ca^2+^ ions compared to the control test (without CaCl_2_).

### Effect of metallic ions and enzyme inhibitors

The results in Table 3 show the effect of Co^2+^, Ba^+2^, Mg^2+^, Ca^2+^, Hg^2+^, Fe^3+^, Na^+^, Mn^2+^, Fe^2+^, Zn^2+^, and Cu^2+^ ions in addition to EDTA on the activity of elastase. Except for Cu^2+^ and Mn^2+^, none of the metal ions tested stimulated the enzyme activity of the elastase. Enzyme activity was inhibited by Ba^2+^, Hg^2+^, Fe^3+^ and Na^+^ but was enhanced by the addition of Cu^2+^ and Mn^2+^ ions. However, EDTA almost wholly repressed the enzymatic activity of the elastase at the tested concentrations.

**Table 3:** Effect of metallic ions and elastase inhibitors.

Metal ions/Inhibitor	Concentration (mM)	Residual activity (%)
Blank	0	100.000
Cu^2+^	5	105.372
Mn^2+^	5	101.438
Mg^2+^	5	90.621
Zn^2+^	5	88.470
Fe^2+^	5	53.145
Co^2+^	5	48.963
Ca^2+^	5	54.634
Hg^2+^	5	37.872
Fe^3+^	5	29.272
Ba^2+^	5	38.572
Na^+^	5	12.602
EDTA	5	0.000
EDTA	10	0.000

### Determination of kinetic parameters of elastase

**Fig 4:**
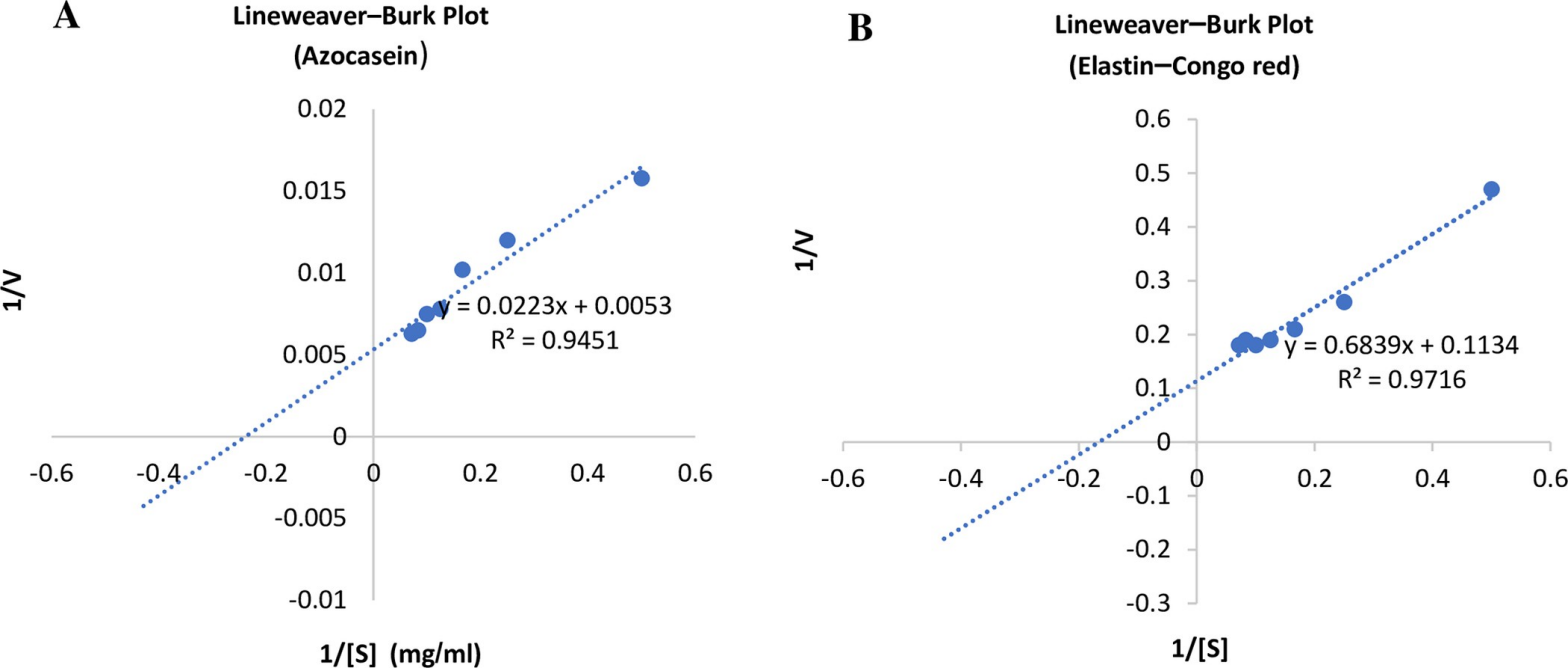
Lineweaver–Burk plot showing the enzyme catalysis against azocasein (Panel A) and elastin–Congo red (Panel B).

The results of the enzyme kinetic study using azocasein and elastin–Congo red as substrates in terms of *K*_m_ and *V*_max_ were calculated from the LB plots (Fig 4). On azocasein, elastase had *K*_m_ and *V*_max_ values of 4.20 mg/mL and 188.67 U/mg, respectively. The *K*_cat_ was 0.232 s^-1^, and the catalytic efficiency (*K*_cat_/*K*_m_) was 0.055 M^-1^ s^-1^. On elastin–Congo red, elastase had *K*_m_ and *V*_max_ values of 6.03 mg/mL and 8.818 U/mg, respectively. The *K*_cat_ was 0.27 s^-1^, and the catalytic efficiency was 0.046 M^-1^ s^-1^.

### Antibacterial activity of elastase

The results of the well diffusion method showed that the tested elastase exhibited antibacterial activity against most of the tested clinical isolates, including *S*. *aureus* subsp. aureus ATCC BAA-1026 (24 mm), *S*. *aureus* subsp. aureus Rosenbach ATCC 25923 (26 mm), *S*. *aureus* subsp. aureus ATCC 43300 (23 mm), *S*. *aureus* subsp. aureus ATCC 29213 (24 mm), *S*. *aureus* ATCC-BAA-976 (19 mm), *Salmonella enterica* subsp. Enterica serovar Enteritidis ATCC 13076 (14 mm), *Salmonella* spp. (15 mm), and *Shigella boydii* ATCC 9207 (29 mm). However, the elastase showed no antibacterial activity against *E*. *coli* ATCC 8739, *E*. *coli* ATCC 25922, *P*. *aeruginosa* ATTC 27853, and *E*. *coli* ATCC 35218. The MICs of elastase determined by broth dilution method against *S*. *boydii* ATCC 9207 and *S*. *aureus* subsp. aureus Rosenbach ATCC 25923 were 37.5 U and 18.75 U, respectively (S5 Fig). The antibacterial activity of elastase against *S*. *boydii* ATCC 9207 and *S*. *aureus* subsp. aureus Rosenbach ATCC 25923 is presented in S6 Fig.

**Fig 5:**
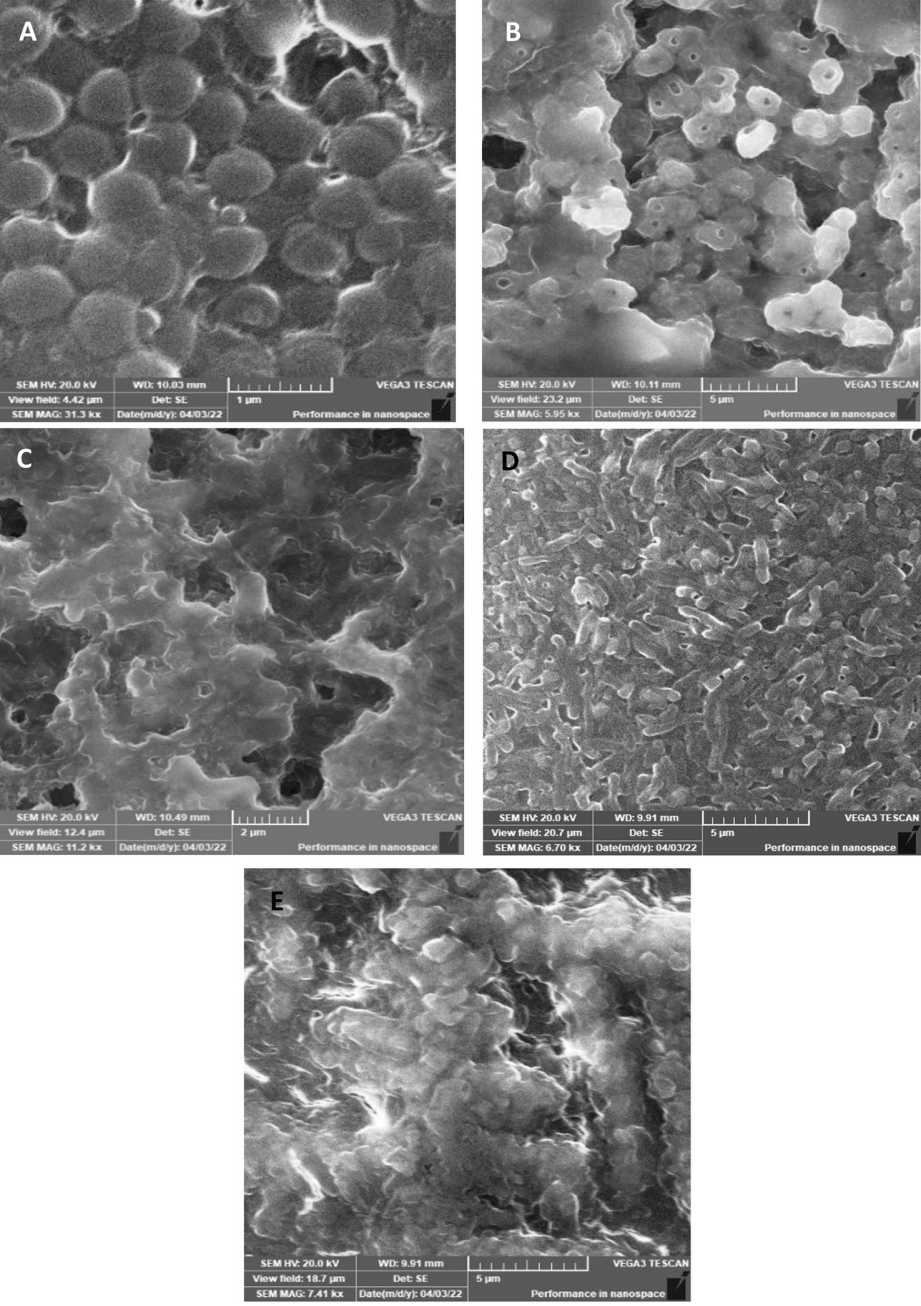
SEM micrographs of the Gram-positive pathogenic bacterium *S*. *aureus* subsp. aureus Rosenbach ATCC 25923 (Panel A represents the control while Panels B and C represent the treated cells) and the Gram-negative pathogenic bacterium *S*. *boydii* ATCC 9207 (Panel D represents the control, while Panel E represents the treated cells).

The SEM study on the most affected harmful bacteria showed that most cells presented damage and perforation. Some cells were observed to have reduced size, and the majority appeared to be fused and melted. These images show the loss of shape and integrity, which led to cell death (Fig 5).

### *In vitro* application of elastase in the degradation of elastin fibers

**Fig 6:**
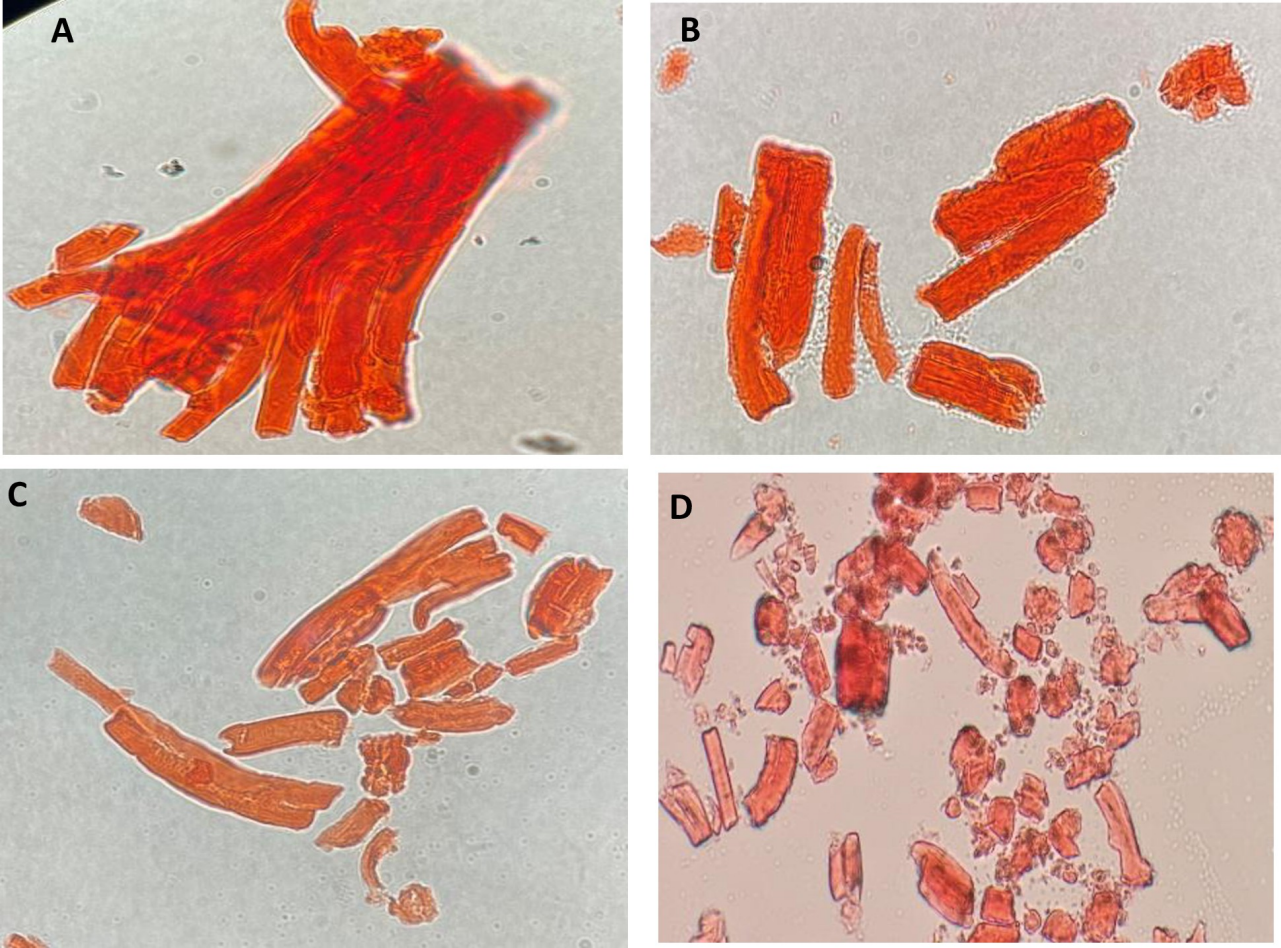
Elastin fibers degradation by gasm32 elastase under a 100x L.M lens. (Panel A: control treatment, Panel B: enzyme treatment for 1 hour, Panel C: enzyme treatment for 2 hours, and Panel D: enzyme treatment for 3 hours).

LM images show multiple longitudinal and transverse crevices on the elastin fiber surface after 1 hour of treatment with the tested elastase (Fig 6B). The size and number of crevices expanded with time (Fig 6C). After 3 hours of enzymatic treatment, fibers were fractured lengthwise by gradual longitudinal fractures and broken into pieces by progressive transverse cracks (Fig 6D).

**Fig 7:**
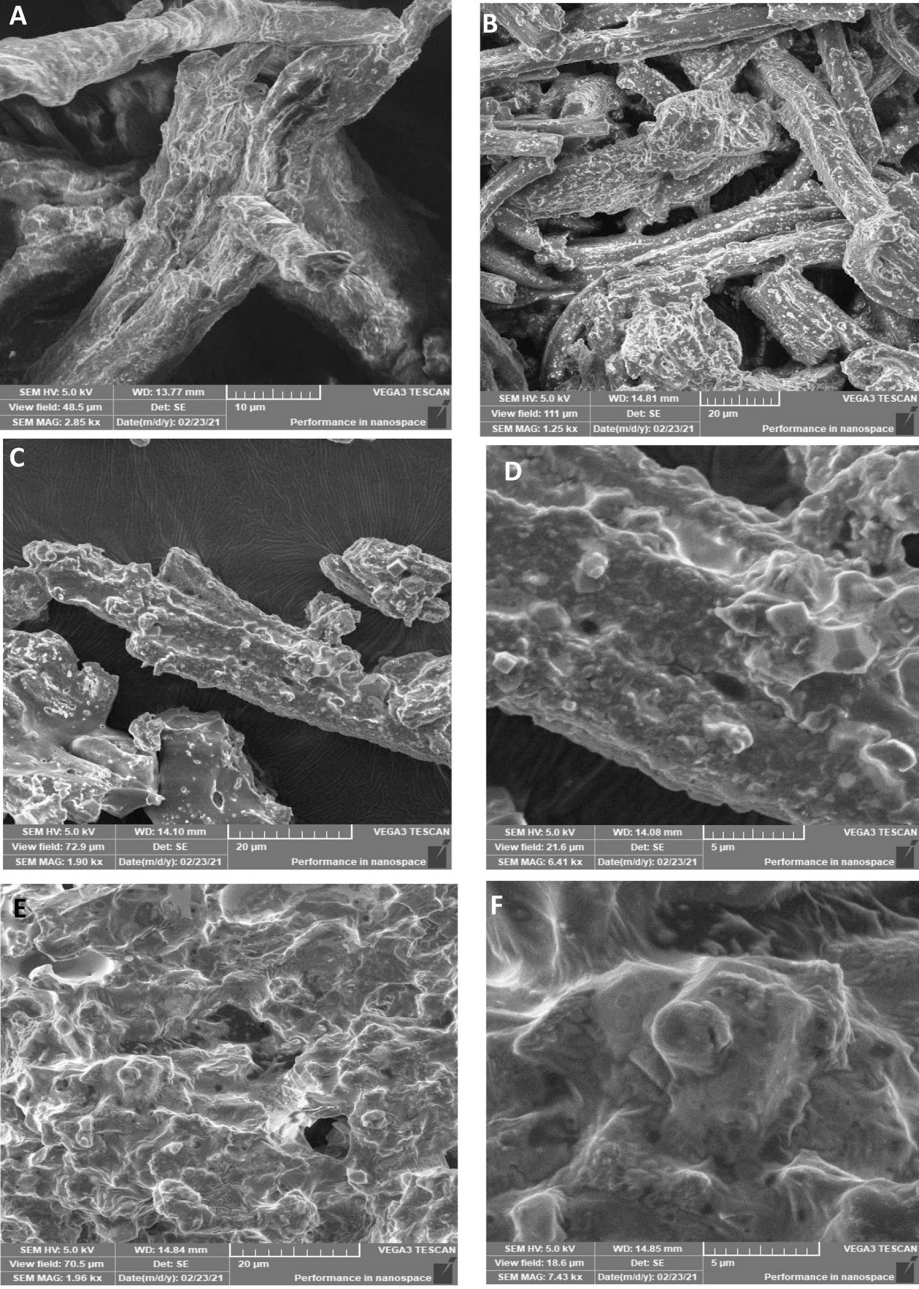
SEM micrographs of elastin degradation by the target elastase (Panel A represents the control, Panel B represents 1 hour of elastase treatment, Panels C and D represent 2 hours of elastase treatment, and Panels E and F represent 3 hours of elastase treatment).

SEM micrographs also show the time-dependent progressive breakdown of elastin fibers exposed to elastase. Fig 7 shows the dramatic strategy by which the fibers were degraded. Cavities were observed on the surface of elastic fibers after 1 hour of treatment (Fig 7B), which expanded gradually in depth and width as the exposure time increased (Fig 7C and 7D). After 3 hours, intact elastin fibers disappeared, leaving irregular pieces in the field of view (Fig 7E and 7F).

## Discussion

Bacterial elastases are mainly grouped under the metalloproteinase family [21]. Indeed, screening for bacteria with elastolytic activity is a direct method for obtaining elastases with desired characteristics. In this study, we screened for bacteria-producing elastases in different localities of Saudi Arabia. The broader sample size and the several sampling locations led to more positive isolation of enzyme producers. However, there are some difficulties in the isolation of bacteria adapted to grow under extreme environments of pressure, pH, temperature, and/or in environments with high concentrations of metals.

Tsai et al. [25] isolated alkaline elastase extracellularly produced by the alkalophilic *Bacillus* strain Ya-B from soil collected around the Tokyo area. Clark et al. [26] isolated a unique alkaline elastase from *Micrococcus luteus* from human skin in the late exponential and stationary growth phases. He et al. [27] obtained elastase from *Bacillus* sp. EL31410 from the soil at a meat-processing factory in Hangzhou, China. Qihe et al. [28] isolated an elastase from *Bacillus* sp. EL31410, which is a good meat tenderizer. This elastase could be a promising substitute for papain as a good meat tenderizer. Malanicheva et al. [29] isolated *Bacillus megaterium* from leached chernozem soil (Kras-nodar krai). Kotb [30] isolated *Bacillus megaterium* KSK-07 from kishk, a traditional Egyptian fermented food. Uttatree et al. [31] isolated *Bacillus megaterium* from marine sediment in the Gulf of Thailand at a depth of 24 meters. Lei et al. [3] isolated a novel elastase from *Chryseobacterium indologenes* from the mud of a meat market in Ya’an city. Elastase is expected to have potential application in meat tenderization because of its specific hydrolysis toward elastin. Kotb et al. [13] reported that among a total of 120 clinical specimens that were collected from the hospitals of Zagazig University (Zagazig, Egypt) from different sources, including pus, urine, wounds, sputum, eyes, blood, and ear infections, only 54 isolates of *Pseudomonas aeruginosa* ZuhP13 were positive for cytotoxic serine-elastase productivity. Zupetic et al. [32] isolated *Pseudomonas aeruginosa* from 238 unique ICU patients belonging to two tertiary-care centers inside the University of Pittsburgh Medical Center health system, and 75% of the isolates produced elastase activity.

The most potent isolate was gasm32, and it was identified molecularly as *P*. *megaterium*. It showed excellent hydrolytic activity against both casein and elastin (S1 Fig). The selected isolate had an elastase productivity of 771.3 U/mL (7.713 A_495_) (Table 1), which is better than the values found by Zins et al. [33] for *P*. *aeruginosa* PAO1 (0.39 A_495_), *P*. *aeruginosa* VMS1120 (0.20 A_495_), *P*. *aeruginosa* VMS7931 (0,109 A_495_), and *C*. *violaceum* 98–9187 (1.09 A_495_), and the values found by Olson and Ohman [34] for *P*. *aeruginosa* PAO1 (0.06 A_495_), *P*. *aeruginosa* FRD2 (0.13 A_495_), and *P*. *aeruginosa* DG1 (0.14 A_495_).

The secreted elastase from *P*. *megaterium* gasm32 was purified to electrophoretic homogeneity and produced a prominent band on SDS‒PAGE indicating a 30 kDa molecular mass (Fig 1). Among other investigators, a 34 kDa molecular mass was reported for an elastase from *P*. *aeruginosa* [35], a 26 kDa molecular mass was reported for an elastase from *C*. *indologenes* [3], and a 23.7 kDa molecular mass was reported for an elastase from the alkalophilic *Bacillus* strain Ya-B [25].

The characteristic properties of the elastases were investigated to achieve the highest activity and determine to which family they belonged. The best reaction pH for gasm32 elastase was determined to be 8.0 (Fig 2), which is the same as that of *P*. *aeruginosa* [36] and the *B*. *megaterium*-TK1 strain isolated from saltwater [37]. This optimal pH was different from those obtained for other elastases in other studies [38], such as the alkaline elastase from *Micrococcus luteus* (pH 9.3, [26]), *B*. *megaterium* (pH 7.5, [29]), *P*. *aeruginosa* ZuhP13 (pH 7.5, [13]), and the alkalophilic *Bacillus* strain Ya-B (pH 11.75, [25]).

The pH stability of gasm32 elastase was observed at pH 6.0 to 10.0 for 2 hours (Fig 2). A wide pH stability spectrum was also observed for the elastase of *C*. *indologenes* (pH 5.0–10.5, [3]), the elastase of *Flavobacterium odoratum* (pH 5.0–10.0, [39]), and the serine-elastase of *P*. *aeruginosa* ZuhP13 (pH range of 6.0–9.0, [13]). The high alkaline character of gasm32 elastase revealed by its activity and stability over a wide pH range also suggests its suitability for use under alkaline conditions such as detergent additives.

The optimum reaction temperature for the target elastase was found to be 45°C (Fig 3A). In reviewing the findings of other investigators, 37°C was the optimal reaction temperature for the elastase of *C*. *indologenes* [3], 40°C was the optimal reaction temperature for the serine-elastase of *P*. *aeruginosa* ZuhP13 [13], the temperature range of 57–59°C was optimal for the elastase of *Micrococcus luteus* [26], and 60°C was the optimal reaction temperature for *Bacillus* Ya-B elastase [25].

Our findings showed that gasm32 elastase was thermally stable for 45 min at 50°C (Fig 3B). Thus, it is more heat stable than the elastase of *Flavobacterium odoratum* (70% inhibition at 50°C for 4 hours, [39]) and the elastase of *C*. *indologenes* (50% inhibition at 55°C, [3]). Our elastase was more heat-sensitive than the serine-elastase of *P*. *aeruginosa* ZuhP13, which was inhibited 50% at 60°C after 30 min of exposure [13]. These differences may be due to the source of the isolate and the type of bacteria.

Similar to other proteases, our elastase was heat-stabilized in the presence of calcium ions [34]. When 15 mM CaCl_2_ was added, 90% elastase activity was sustained for 15 min at 60°C (Fig 3D), while there was a 25% decrease in elastase activity in the absence of Ca^2+^ ions. Moreover, it maintained 51% of its original activity after 60 min in the presence of Ca^2+^ while in the absence of Ca^2+^ it retained 3% of its initial activity. The presence of Ca^2+^ ions also increases the thermal stability of a protease from *B*. *subtilis* FBL-1 [40] and the neutral proteases of *B*. *megaterium* AU02 [16]. The increased enzyme stability in the presence of calcium ions may be due to the ability of calcium ions to act as an ionic bridge between carboxylic groups of amino acids forming the enzyme molecules, thereby retaining the three dimensions of the enzyme [41]. Additionally, it could be explained by enhancing protein‒protein interactions and associating Ca^2+^ with autolysis sites to avoid autolysis and thermal unfolding [42].

The impact of various cations on the activity of *P*. *megaterium* gasm32 elastase varied significantly (Table 3). Cu^2+^ and Mn^2+^ ions stimulated enzyme activity, whereas Mg^2+^, Zn^2+^, Fe^2+^, Co^2+^, Ca^2+^, Hg^2+^, Fe^3+^, Ba^2+^, and Na^+^ ions decreased it. Comparable results were reported by Manavalan et al. [37], who found that the enzyme activity of *B*. *megaterium*-TK1 increased in the presence of Mn^2+^ ions, while it decreased in the presence of Zn^2+^ and Hg^2+^ ions. On the other hand, Abd El-Aziz and Hassan [9] found that the elastase from *B*. *subtilis* was completely inhibited by Zn^2+^ and Mn^2+^. However, Kotb et al. [13] found that the activity of *P*. *aeruginosa* serine elastase was not affected in the presence of Hg^2+^, Cu^2+^, Zn^2+^, Co^2+^, Ca^2+^, Mn^2+^, Mg^2+^, Ba^2+^, and Fe^3+^ ions.

The gasm32 enzyme was completely inhibited by EDTA, indicating that it is most likely a metalloprotease. Similar findings were found by Zins et al. [33] and Abd El-Aziz and Hassan [9]. EDTA did not affect enzymatic activity in the study of Kotb et al. [13], as the enzyme they studied was a serine elastase. Metals, especially divalent cations, are essential for some enzymes’ catalytic activity and/or stability because they may act as cofactors and frequently serve as salt or ion bridges among nearby amino acid residues. EDTA is a metal–ion chelating agent that binds divalent metal ions. Therefore, it can inactivate metal ion-requiring enzymes (metalloproteases) [43]. In general, elastases of bacteria such as *B*. *amyloliquefaciens*, *B*. *subtilis*, *Pseudomonas* sp., *E*. *coli*, and *Lysobacter enzymogenes* are either serine proteases or metalloproteases, while aspartic proteases and cysteine proteases are mostly found in higher organisms such as animals and plants [44].

The data from LB plots (Fig 4) revealed that the elastase of *P*. *megaterium* gasm32 has a catalytic efficiency against azocasein of 0.055 M^-1^ s^-1^, a *K*_m_ of 4.20 mg/mL, and a *K*_cat_ of 0.232 s^-1^. In addition, its catalytic efficiency against elastin–Congo red was 0.046 M^-1^ s^-1^, its *K*_m_ was 6.03 mg/mL, and its *K*_cat_ was 0.27 s^-1^. Given previous research, the catalytic efficiency of the serine-elastase of *P*. *aeruginosa* ZuhP13 was 4.62 M^-1^ s^-1^, its *K*_m_ was 1.32 mg/mL, and its *K*_cat_ was 1.27 s^-1^ [13]. The *K*_m_ of the protease from *B*. *Megaterium* AU02 using azocasein was 0.722 mg/mL, and the *V*_max_ was 0.018 U/mg [16].

Antibacterial agents could benefit from the proteolytic potential of bacterial elastases to inhibit MDR pathogenic bacteria. This research provided beneficial results as a step toward developing bioactive peptide compounds. The tested elastase exerted excellent antibacterial activity against well-known antibiotic-resistant bacteria, especially *S*. *boydii* ATCC 9207 (Fig 5 and S6 Fig). This bacterium is a Gram-negative type that causes diarrhea (often bloody), fever, and stomach cramps. Antibiotic resistance among *Shigella* spp. is increasing globally (Center for Diseases Control—CDC, Atlanta, USA).

Moreover, the gasm32 elastase strongly inhibited *S*. *aureus* subsp. aureus Rosenbach ATCC 25923 (Fig 5 and S6 Fig). This pathogen is a Gram-positive bacterium related to food poisoning, respiratory diseases, and skin infections. *S*. *aureus* has obtained numerous strategies to develop resistance to almost all antibiotics. Hence, antibiotic treatment is not always successful [45]. Similarly, elastase showed activity against *Salmonella* spp., a common Gram-negative human pathogen. The majority of *Salmonella* strains cause salmonellosis. Other *Salmonella* strains cause typhoid fever or paratyphoid fever. *Salmonella s* resistance to essential antibiotics has increased, limiting treatment options for people with severe infections (Center for Diseases Control—CDC, Atlanta, USA).

SEM analysis of the most affected bacteria, *S*. *boydii* and *S*. *aureus* (Fig 5), revealed that cells became fused and malformed with surface shrinkage. In addition, prominent perforation of cells was very clear in the case of *S*. *aureus* (Fig 5C). Ultimately, these effects might lead to cell death. Regarding other investigators, the alkaline protease produced by *B*. *tequilensis* ZMS-2 was found to be effective against human pathogens such as *S*. *aureus* (27 mm inhibition zone), *Klebsiella pneumonia* (17 mm), *E*. *coli* (15 mm), and *B*. *licheniformis* (20 mm) [21].

The *in vitro* application of gasm32 elastase in the degradation of macroscopic insoluble elastin fibers was confirmed using both LM and SEM. Elastin fiber degradation began on the surface of specific areas. This resulted in larger fissures and cavities, as shown by LM (Fig 6) and SEM (Fig 7). Over time, the fissures and cavities on the elastin fiber surface expanded, causing the fiber to break into small fragments, which were then further broken down into tiny peptides and amino acid residues [46].

## Conclusion

In conclusion, an elastase with good characteristics was recovered and purified from the local bacterium *P*. *megaterium* gasm32. The productivity was greatly maximized after optimizing the physical and nutritional conditions (unpublished data). Additionally, the factors affecting its activity and stability were studied. Our study confirmed that it is not only able to hydrolyze elastin efficiently but also has antibacterial activity, including activity against skin burn pathogens. Therefore, it may be a candidate for regenerating and healing elastin fibers of damaged skin with antibacterial activity against contaminating bacteria. For future work and perspective, it is recommended to examine its mode of action and safety for industrialization and commercialization before it is used as a commercial pharmaceutical product.

## Supporting information

S1 Fig Protease activity of isolate gasm32 on skimmed milk agar plates (A-B) and elastase activity on nutrient agar- elastin plates (C-D). Panels a and c represent the spot tests, while Panels b and d represent the well tests after 24 hours of incubation at 37°C. In the well test, 100 μl of crude enzyme was added into each 7 mm diameter well.
(DOCX)

S2 Fig Agarose gel electrophoresis showing the 700 bp PCR amplicons for *P*. *megaterium* gasm32, *B*. *aryabhattai* gasm34, *K*. *pneumoniae* gasm37, *Serratia marcescens* gasm82, *Serratia marcescens* gasm91, *Macrococcus caseolyticus* gasm25, *Proteus mirabilis* gasm43, *Proteus sp*. gasm71, *Enterobacter cloacae* gasm27, and *Lactococcus lactis* gasm28. The positive control was *E*. *coli* BUN001, while the negative control consisted of the master mix with deionized sterile water. DNA was extracted using the QIAGEN kit. L contained a DNA marker.
(DOCX)

S3 Fig A neighbor-joining phylogenetic tree for *P*. *megaterium gasm32*. The sequence retrieved is marked. The bootstrap value is indicated by the scale bar.
(DOCX)

S4 Fig The previous version of SDS-PAGE of the purified elastase using 5% stacking gel and 15% separating gel. (DOCX)

S5 Fig The MIC of elastase determined by broth dilution method against: (A) *S*. *aureus* subsp. aureus Rosenbach ATCC 25923 was 18.75 U/ml, (B) *S*. *boydii* ATCC 9207 was 37.5 U/ml.
(DOCX)

S6 Fig Antibacterial activity of elastase against pathogenic strains: (A) S. boydii ATCC 9207, (B) S. aureus subsp. aureus Rosenbach ATCC 25923.
(DOCX)

## Abbreviations

BSA: Bovine serum albumin
EDTA: Ethylene diamine tetraacetic acid
*K*_cat_: Catalytic rate constant
*K*_m_: Michaelis–Menten constant
LB broth: Luria-Bertani broth
LB plot: Lineweaver–Burk plot
LM: Light microscope
MDR: Multidrug resistance
MHB: Muller Hinton Broth
SEM: Scanning electron microscopy
TCA: Trichloroacetic acid
*V*_max_: Maximum velocity
