# Supplementary material for: A survey of elastase-producing bacteria and characteristics of the most potent producer, Priestia megaterium gasm32
Source: PLoS One. 2023 Mar 13;18(3):e0282963. doi: 10.1371/journal.pone.0282963 (PMC10010523; doi:10.1371/journal.pone.0282963)
Supplement: S1 Fig — Protease activity of isolate gasm32 on skimmed milk agar plates (A-B) and elastase activity on nutrient agar- elastin plates (C-D). Panels a and c represent the spot tests, while Panels b and d represent the well tests after 24 hours of incubation at 37°C. In the well test, 100 μl of crude enzyme was added into each 7 mm diameter well. (DOCX) [file pone.0282963.s001.docx]

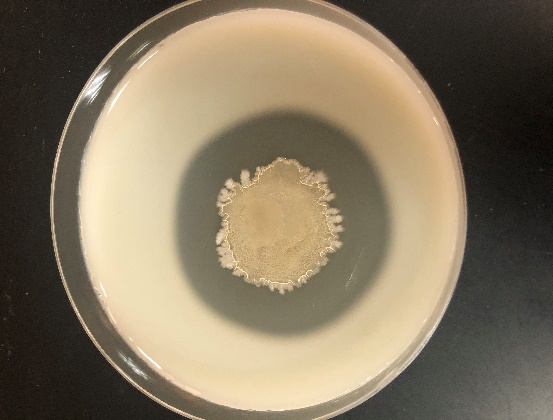


**A**


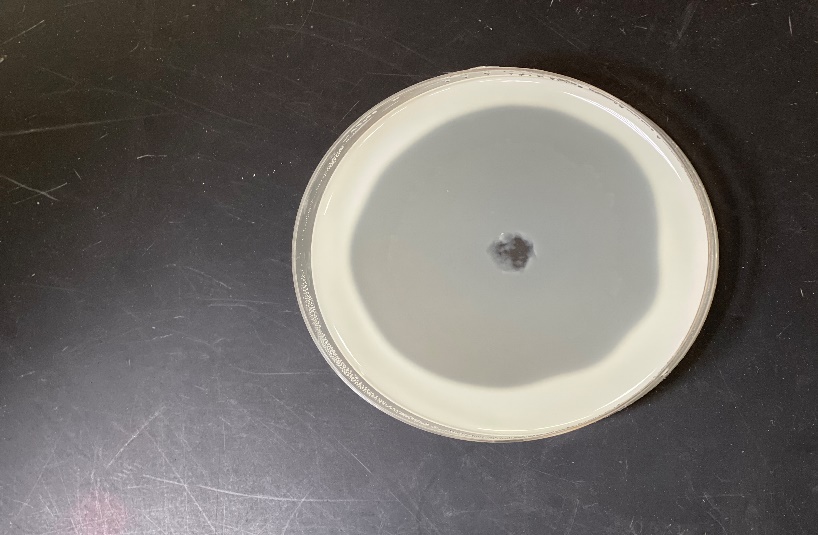


**B**


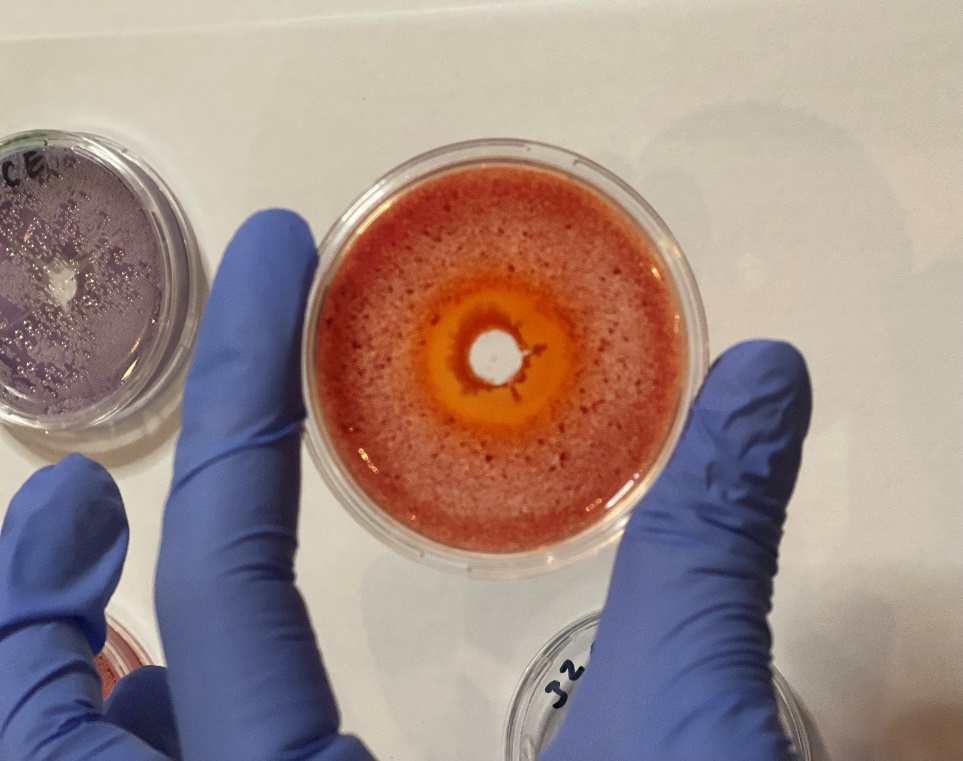


**D**


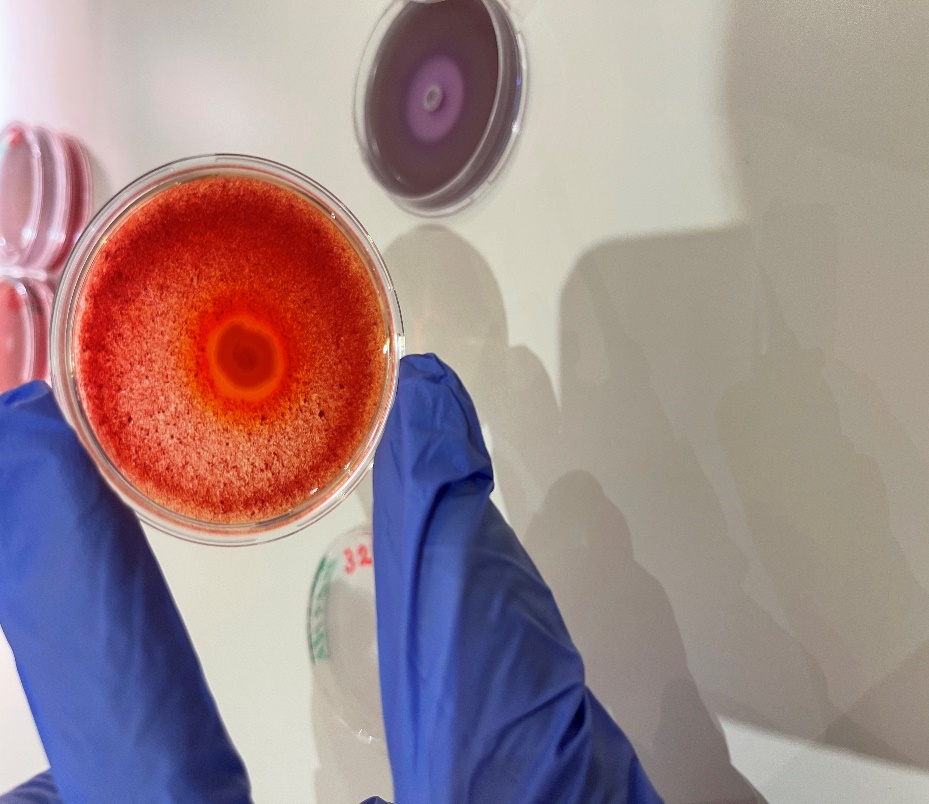


**C**

**S1 Fig.** **Protease activity of isolate gasm32 on skimmed milk agar plates (A-B) and elastase activity on nutrient agar- elastin plates (C-D). Panels a and c represent the spot tests, while Panels b and d represent the well tests after 24 hours of incubation at 37 °C. In the well test, 100 µl of crude enzyme was added into each 7 mm diameter well.**
