# Supplementary material for: A survey of elastase-producing bacteria and characteristics of the most potent producer, Priestia megaterium gasm32
Source: PLoS One. 2023 Mar 13;18(3):e0282963. doi: 10.1371/journal.pone.0282963 (PMC10010523; doi:10.1371/journal.pone.0282963)
Supplement: S2 Fig — The positive control was E. coli BUN001, while the negative control consisted of the master mix with deionized sterile water. DNA was extracted using the QIAGEN kit. L contained a DNA marker. (DOCX) [file pone.0282963.s002.docx]

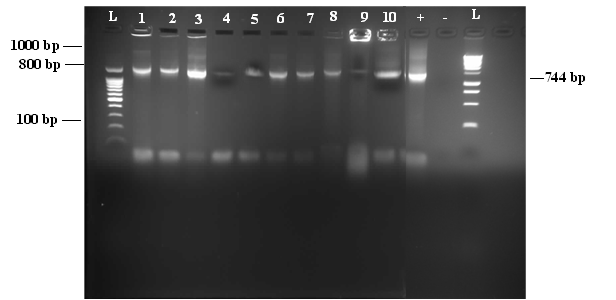


**L**

**S2 Fig. Agarose gel electrophoresis showing the 700 bp PCR amplicons for P. megaterium gasm32, B. aryabhattai gasm34, K. pneumoniae gasm37, Serratia marcescens gasm82, Serratia marcescens gasm91, Macrococcus caseolyticus gasm25, Proteus mirabilis gasm43, Proteus sp. gasm71, Enterobacter cloacae gasm27, and Lactococcus lactis gasm28. The positive control was E. coli BUN001, while the negative control consisted of the master mix with deionized sterile water. DNA was extracted using the QIAGEN kit. L contained a DNA marker.**
