# Supplementary material for: A survey of elastase-producing bacteria and characteristics of the most potent producer, Priestia megaterium gasm32
Source: PLoS One. 2023 Mar 13;18(3):e0282963. doi: 10.1371/journal.pone.0282963 (PMC10010523; doi:10.1371/journal.pone.0282963)
Supplement: S3 Fig — The sequence retrieved is marked. The bootstrap value is indicated by the scale bar. (DOCX) [file pone.0282963.s003.docx]

**S3 Fig. A neighbor-joining phylogenetic tree for *P. megaterium gasm32.*** **The sequence retrieved is marked. The bootstrap value is indicated by the scale bar.**
