# Supplementary material for: A survey of elastase-producing bacteria and characteristics of the most potent producer, Priestia megaterium gasm32
Source: PLoS One. 2023 Mar 13;18(3):e0282963. doi: 10.1371/journal.pone.0282963 (PMC10010523; doi:10.1371/journal.pone.0282963)
Supplement: S4 Fig — (DOCX) [file pone.0282963.s004.docx]

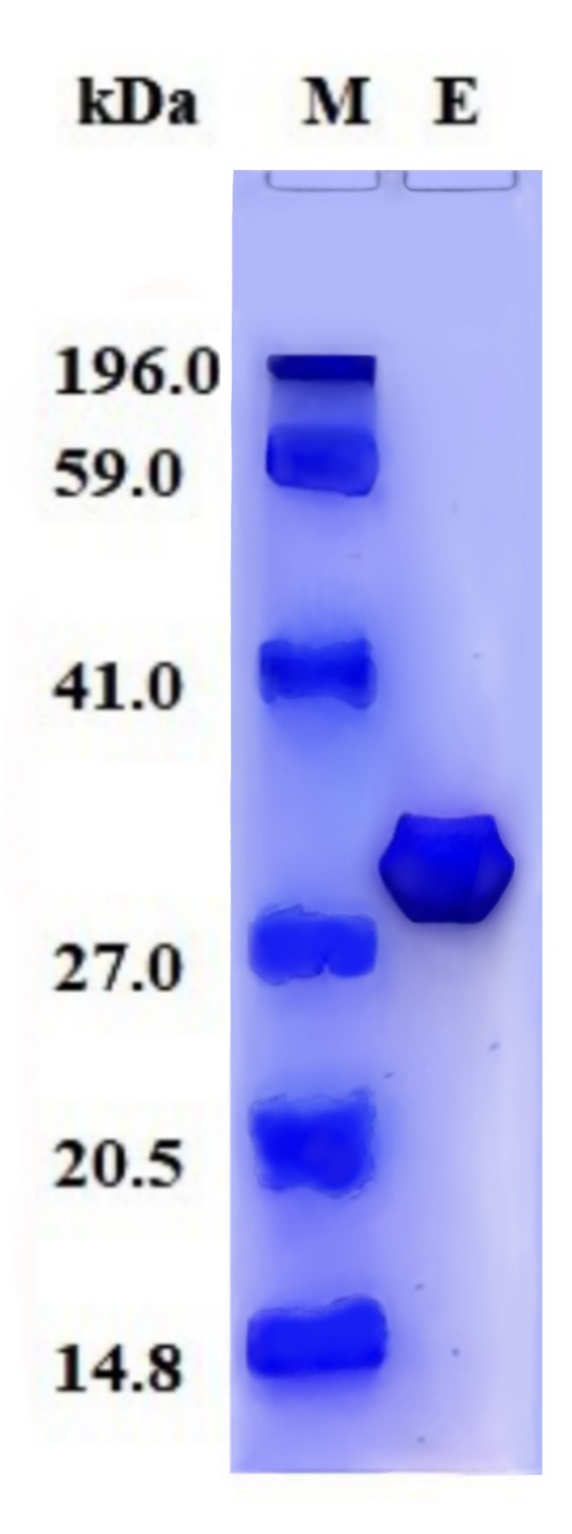


**S4 Fig. The previous version of SDS-PAGE of the purified elastase using 5% stacking gel and 15% separating gel.**
