# Supplementary material for: A survey of elastase-producing bacteria and characteristics of the most potent producer, Priestia megaterium gasm32
Source: PLoS One. 2023 Mar 13;18(3):e0282963. doi: 10.1371/journal.pone.0282963 (PMC10010523; doi:10.1371/journal.pone.0282963)
Supplement: S5 Fig — The MIC of elastase determined by broth dilution method against: (A) S. aureus subsp. aureus Rosenbach ATCC 25923 was 18.75 U/ml, (B) S. boydii ATCC 9207 was 37.5 U/ml. (DOCX) [file pone.0282963.s005.docx]

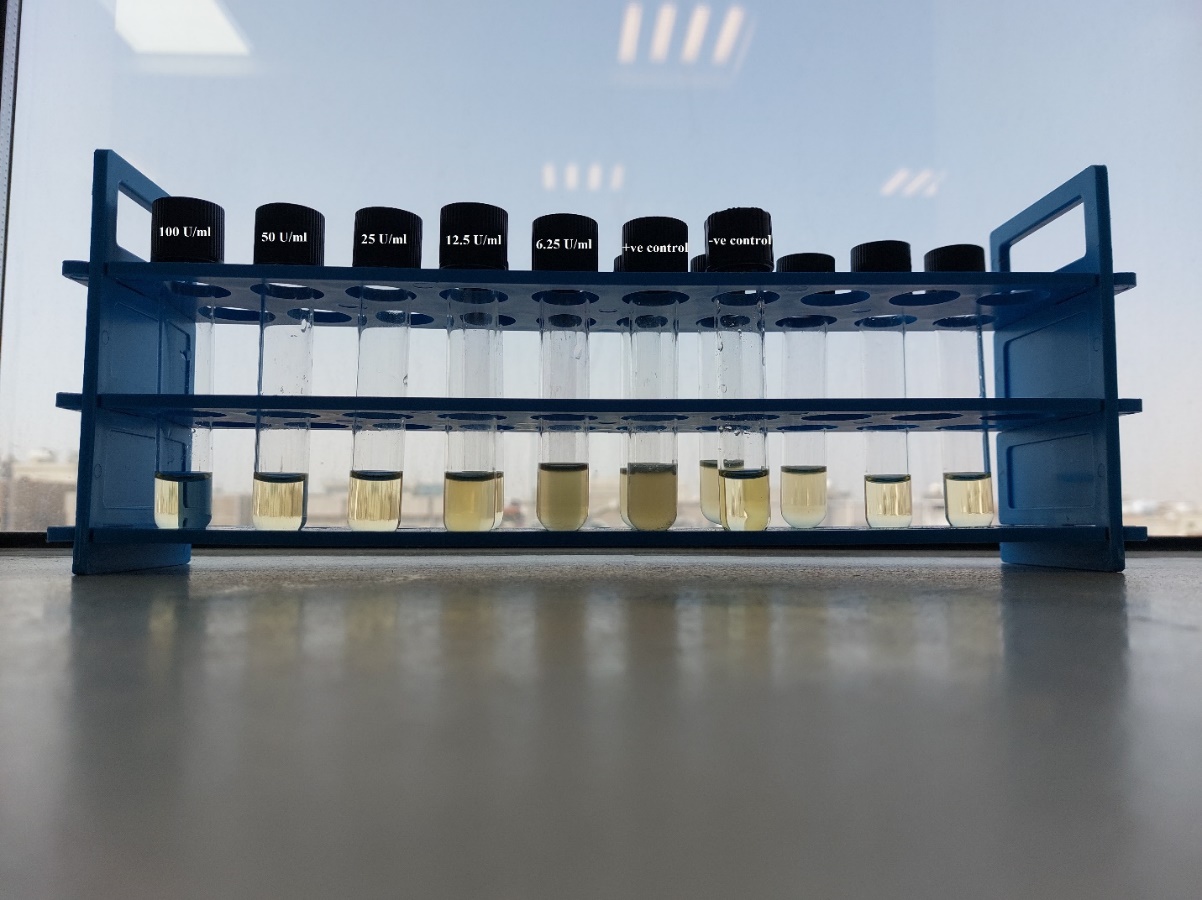


**A**


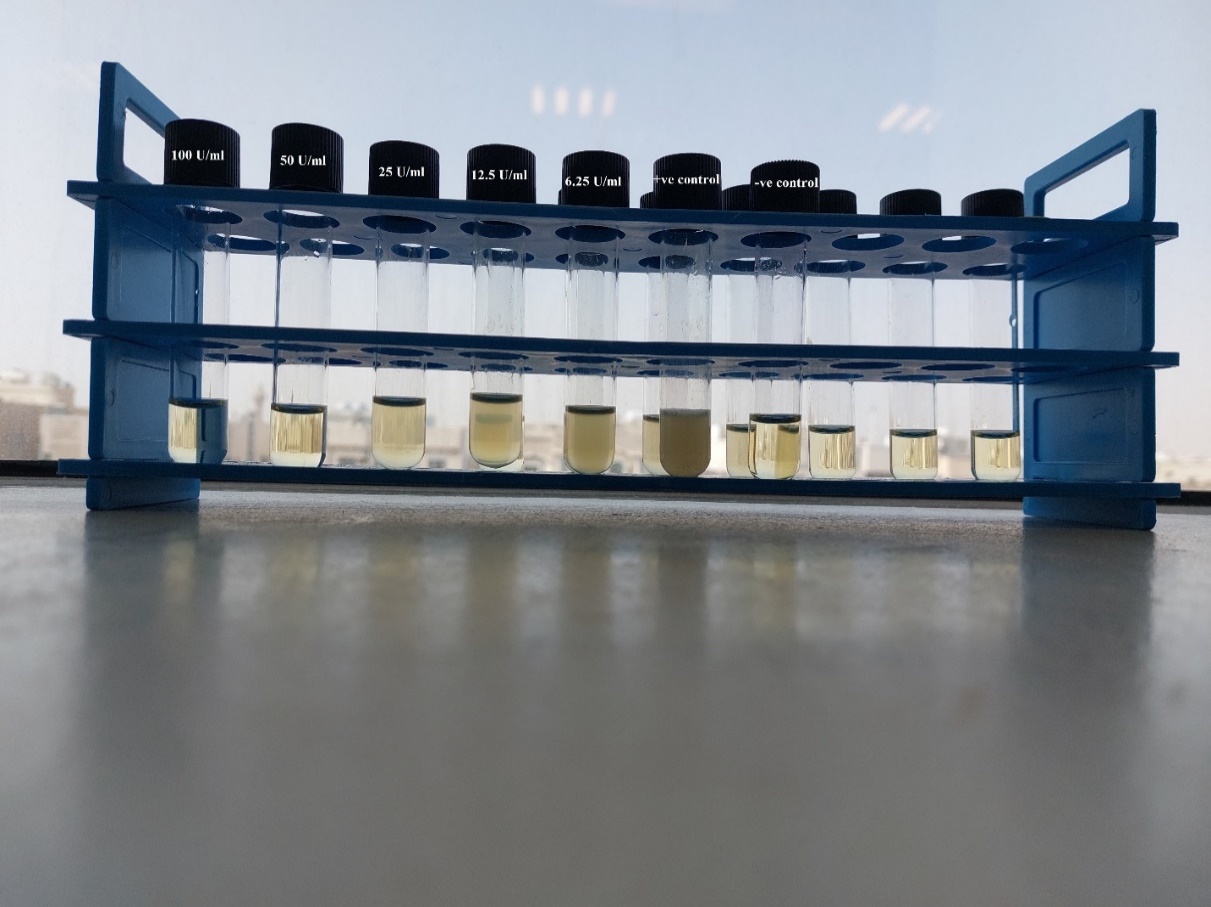


**B**

**S5 Fig. The MIC of elastase determined by broth dilution method against:** (**A**) **S. aureus subsp. aureus Rosenbach ATCC 25923 was 18.75 U/ml, (B) S. boydii ATCC 9207 was 37.5 U/ml.**
