# Supplementary material for: A survey of elastase-producing bacteria and characteristics of the most potent producer, Priestia megaterium gasm32
Source: PLoS One. 2023 Mar 13;18(3):e0282963. doi: 10.1371/journal.pone.0282963 (PMC10010523; doi:10.1371/journal.pone.0282963)
Supplement: S6 Fig — Antibacterial activity of elastase against pathogenic strains: (A) S. boydii ATCC 9207, (B) S. aureus subsp. aureus Rosenbach ATCC 25923. (DOCX) [file pone.0282963.s006.docx]

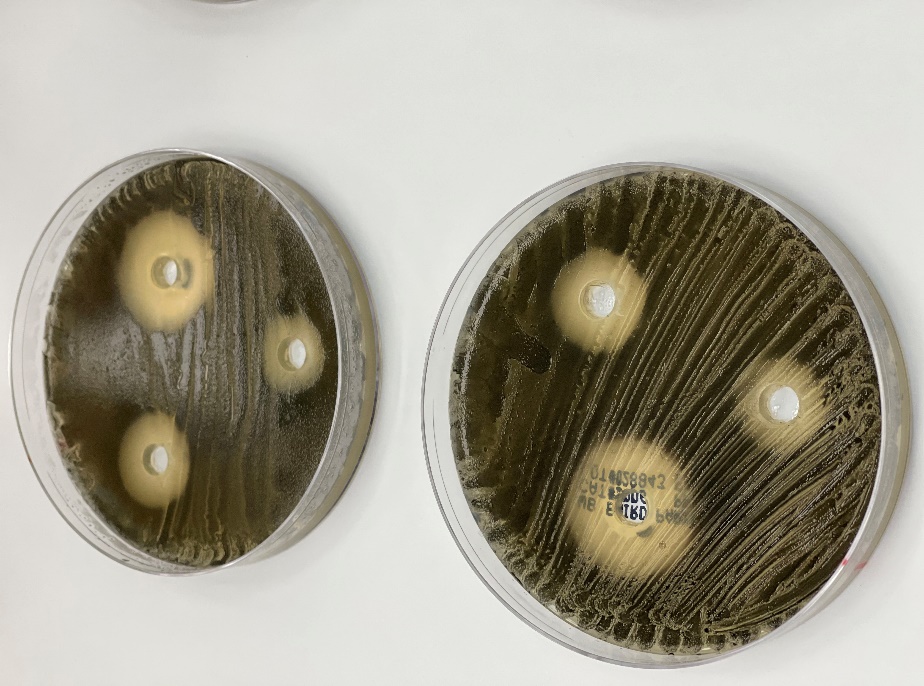

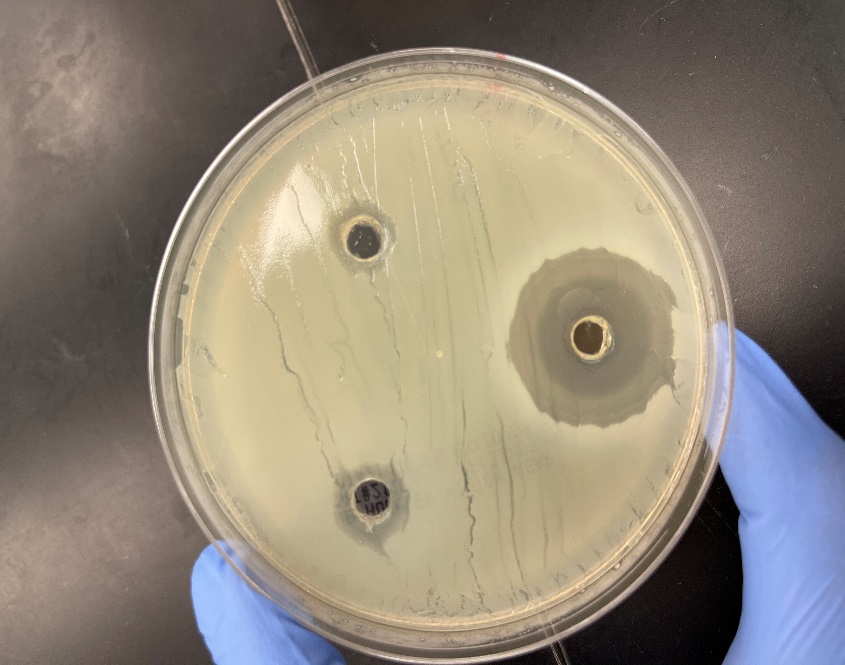


**B**

**A**

**S6 Fig. Antibacterial activity of elastase against pathogenic strains:** (**A**) **S. boydii ATCC 9207, (B) S. aureus subsp. aureus Rosenbach ATCC 25923. Three concentrations of enzyme were used against the tested bacteria (100 U, 50 U, and 25 U).**
